# Evaluation of easily measured risk factors in the prediction of osteoporotic fractures

**DOI:** 10.1186/1471-2474-6-47

**Published:** 2005-09-05

**Authors:** Robert Bensen, Jonathan D Adachi, Alexandra Papaioannou, George Ioannidis, Wojciech P Olszynski, Rolf J Sebaldt, Timothy M Murray, Robert G Josse, Jacques P Brown, David A Hanley, Annie Petrie, Mark Puglia, Charlie H Goldsmith, W Bensen

**Affiliations:** 1 Medical Science, McMaster University, Hamilton, Ontario, Canada; 2 Medicine, McMaster University, Hamilton, Ontario, Canada; 3 Medicine, University of Saskatchewan, Saskatoon, Saskatchewan, Canada; 4 Medicine, University of Toronto, Toronto, Ontario, Canada; 5 Medicine, Laval University, Ste-Foy, Quebec, Canada; 6 Medicine, University of Calgary, Calgary, Alberta, Canada

## Abstract

**Background:**

Fracture represents the single most important clinical event in patients with osteoporosis, yet remains under-predicted. As few premonitory symptoms for fracture exist, it is of critical importance that physicians effectively and efficiently identify individuals at increased fracture risk.

**Methods:**

Of 3426 postmenopausal women in CANDOO, 40, 158, 99, and 64 women developed a new hip, vertebral, wrist or rib fracture, respectively. Seven easily measured risk factors predictive of fracture in research trials were examined in clinical practice including: age (**<65**, 65–69, 70–74, 75–79, 80+ years), rising from a chair with arms (yes, **no**), weight (< 57, **≥ 57**kg), maternal history of hip facture (yes, **no**), prior fracture after age 50 (yes, **no**), hip T-score (**>-1**, -1 to >-2.5, ≤-2.5), and current smoking status (yes, **no**). Multivariable logistic regression analysis was conducted.

**Results:**

The inability to rise from a chair without the use of arms (3.58; 95% CI: 1.17, 10.93) was the most significant risk factor for new hip fracture. Notable risk factors for predicting new vertebral fractures were: low body weight (1.57; 95% CI: 1.04, 2.37), current smoking (1.95; 95% CI: 1.20, 3.18) and age between 75–79 years (1.96; 95% CI: 1.10, 3.51). New wrist fractures were significantly identified by low body weight (1.71, 95% CI: 1.01, 2.90) and prior fracture after 50 years (1.96; 95% CI: 1.19, 3.22). Predictors of new rib fractures include a maternal history of a hip facture (2.89; 95% CI: 1.04, 8.08) and a prior fracture after 50 years (2.16; 95% CI: 1.20, 3.87).

**Conclusion:**

This study has shown that there exists a variety of predictors of future fracture, besides BMD, that can be easily assessed by a physician. The significance of each variable depends on the site of incident fracture. Of greatest interest is that an inability to rise from a chair is perhaps the most readily identifiable significant risk factor for hip fracture and can be easily incorporated into routine clinical practice.

## Background

Osteoporosis affects one in four women and one in eight men in Canada [1]. Its consequences are devastating both to the individual and society in terms of suffering, disability, and increased health care expenses. It is expected that over the next 40 years the number of hip fractures alone will increase exponentially, leading to a problem of epidemic proportions [2]. Fragility fractures are the most important and disabling consequence of osteoporosis and result in a loss of functional ability and increased morbidity and mortality. Estimated conservatively, a 50 year old woman has a 40 percent lifetime risk of a hip, vertebral or wrist fracture [3]. In 1993, the Canadian health care system spent more than $1.3 billion on osteoporosis-related fractures [4]. Recent research has shown that the mean one-year cost of a hip fracture, including direct and indirect costs, is (CAN) $26 527 [5].

Predicting incident fractures is critical today, but in light of the aging demographics, will have even greater importance in the future. Early recognition and treatment of osteoporotic patients is crucial to the prevention of these fractures. A number of risk factors have come to define those at increased risk for osteoporotic fracture and much work has been done in an attempt to delineate the critical variables [6-11]. In order for risk assessment to be effective and efficient, it must be practical and have high predictive value for the identification of fractures. Black et al., with use of data from the Study of Osteoporotic Fractures (SOF), developed the Fracture Index [12]. This assessment tool for predicting fracture risk in osteoporotic post-menopausal women was shown to be predictive of incident osteoporotic fractures (hip, vertebral, wrist and rib) in post-menopausal women and was validated using the EPIDOS study [12]. The Fracture Index assessment tool considers seven variables: age, fracture after age 50, history of maternal hip fracture after age 50, weight less than 125 lb (57 kg), smoking status, use of arms to stand up from a chair and bone mineral density (BMD) T-score [12]. These variables were chosen not only based on their predictive value but also because of their ease of assessment in a clinical setting. As a result, we sought to examine the usefulness of these risk factors in a clinical specialist setting.

The purpose of this study was to examine whether the risk factors outlined in the Fracture Index could be used to predict new incident osteoporotic fractures of the hip, vertebra, wrist, and rib in postmenopausal women who are registered in The Canadian Database for Osteoporosis and Osteopenia (CANDOO).

## Methods

### Study design

An analysis using prospective collected patient information from the CANDOO database was conducted. The CANDOO registry is a multi-site (Calgary, Winnipeg, Saskatoon, Toronto, Hamilton, Montreal, Quebec City) database consisting of more than 10,000 men and women who have been referred to specialists for osteoporosis [13]. The CANDOO database is designed to gather osteoporosis-related clinical information in a prospective manner displaying a record at each clinical visit. Patient information is entered and recorded into a central database [13]. It includes information on patient demographics; vertebral, rib, wrist and hip fracture history; gynecological history; use of osteoporosis related drugs; drug side effects; use of corticosteroids and other medications; dietary calcium intake; smoking habits; physical activity; fall history; prior medical history; family history including fractures; a self administered osteoporosis health related quality of life instrument; basic laboratory results and bone density measurements [13]. Patients were followed yearly with detailed assessments at clinical centres. Each yearly visit involved the collection of information similar to that collected at baseline.

All centres with a baseline CANDOO assessment (visit 1) between 1990 and 1999 with follow-up data available were selected. Only postmenopausal women ≥ 55 years of age with follow-up data available entered in CANDOO were considered for inclusion. Individuals with no follow-up assessments or males were excluded. Any individuals experiencing multiple fractures were also excluded in order to group subjects into hip, vertebral, wrist or rib fracture groups respectively. This exclusion criteria limited the number of applicable subjects from CANDOO and simultaneously implicated only females in the analysis. Fractures in CANDOO were determined based on either self-reports or X-ray confirmation.

For the current study we examined the seven easily measured clinical risk factors for fracture outlined in the Fracture Index. Each risk factor was expressed as an independent variable. The major dichotomous risk factors abstracted were: vertebral, rib, wrist or hip fractures after age 50 (yes/no); maternal family history of fracture (yes/no); weight < 57 kg (≤ 57 kg, > 57 kg); smoking status (yes/no); use of arms to stand from seated position (yes/no). Non-dichotomous risk factors abstracted included: age (<65, 65–69, 70–74, 75–79, 80–84, 85+ years); bone mineral density total hip T-score (> -1, -1 to -2.5, ≤ -2.5) (measurements were made using dual energy X-ray absorptiometry using Lunar or Hologic densitometers) [13]. Adjustments for osteoporotic drug treatment and inclusion of the osteoporosis quality of life questionnaire (OQLQ) as a variable were considered.

### Statistical analysis

Using the seven independent variables we conducted multivariate logistic regression analyses for each fracture type to identify which of the seven risk factors were related to fracture in patients from the CANDOO database.

While longitudinal databases are a powerful research tool, a common weakness is that of missing or incomplete data. To minimize the bias associated with missing data, multiple imputation was utilized to replace missing data prior to the analyses [14,15]. In this case, 10 complete data sets were generated. The multiple imputed datasets were then analyzed using standard procedures for complete data and the results were pooled. Odds ratios and the associated 95% confidence intervals were calculated.

## Results

### Baseline characteristics

A total of 3426 patients were evaluated. Of those patients examined, 99 developed a wrist fracture, 64 a rib fracture, 158 a vertebral fracture, 40 a new hip fracture and 3065 experienced no fracture. Individuals in CANDOO who developed multiple fractures, 53 in total, were excluded from the analyses. The mean age and weight of the patients was 68 years and 64 kg, respectively. Mean total hip T-score was -1.9.

Baseline characteristics for the risk factors examined are displayed in Table 1. More than one third (36.8 %) of the women included were <65 years of age. While many subjects did not have a BMD assessment, 54.8 % of those that did had a T-score between -1 and -2.5. Only 5.6 % of postmenopausal women in CANDOO reported having a maternal history of hip fracture while 41.2 % had a prior fracture after the age of 50 years and 10.5 % were current smokers. Of the entire group, approximately half were only able to rise from a chair with the use of arms (as compared with no arms) and 30.2 % of patients weighed less the 57 kg.

**Table 1:** Baseline characteristics of risk factors for patient cohort.

**RISK FACTOR**	**n (% of cohort)**
Age < 64.5 years	1259 (36.8)
Age 64.5–69.5	740 (21.6)
Age 69.5–74.5	751 (21.9)
Age 74.5–79.5	426 (12.4)
Age 79.5–84.5	186 (5.4)
Age ≥ 84.5	64 (1.9)
Total Hip T-score, T > -1	89 (19.9)
Total Hip T-score, -2.5<T<-1	245 (54.8)
Total Hip T-score, T ≤ -2.5	113 (25.3)
Maternal history of hip fracture	192 (5.6)
Prior fracture >50 years	1413 (41.2)
Current smokers	361 (10.5)
Rise from a chair with arms	1242 (49.2)
Weight < 57 kg	867 (30.2)

### Hip fractures

**Figure 1:**
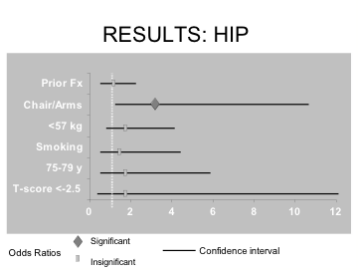
Odds ratios and confidence intervals for each risk factor that predicts higher risk of hip fracture.

The results of multivariable logistic regression analyses for each of the seven risk factors examined to predict a new fracture event in the hip are presented in Table 2. Prior fracture after the age of 50 years, those currently smoking, those between 75–79 years of age and those with a T-score of < -2.5 indicated a trend towards higher risk of developing a new hip fracture. The one factor that was statistically significant for predicting subsequent hip fractures were those individuals only able to rise from a chair with the aid of their arms. These individuals had an approximate odds ratio of 3.6. The 95% confidence interval of each risk factor predicting higher risk of developing a new fracture at the hip site is shown in Figure 1. All the factors had fairly wide confidence intervals, in part due to the overall low incidence of hip fractures. Maternal history of hip fracture was excluded from this model as the hip fracture group contained no data on maternal history of hip fracture.

**Table 2:** Hip Fracture Results Odds ratios and 95% confidence intervals point estimates for each risk factor in the development of new hip fracture

**RISK FACTOR**	**Odds Ratio**	**95% Confidence Interval**
Weight (< 57 Kg) *Reference Level: ≥ 57 Kg	1.70	0.719; 4.037
Use of Arms to Rise *Reference Level: rise without arms	3.58	1.173; 10.927
Smoking Status *Reference Level: no smoking	1.48	0.485; 4.536
Age (65 to 69 y) *Reference Level: < 65	1.53	0.505; 4.667
Age (70–74 y) *Reference Level: < 65	0.98	0.280; 3.421
Age (75–79 y) *Reference Level: < 65	1.62	0.451; 5.840
Age (80–84 y) *Reference Level: < 65	1.52	0.296; 7.780
Age (85+y) *Reference Level: < 65	4.36	0.823; 23.078
Previous Fracture After 50 y *Reference Level: no fracture after 50	1.08	0.477; 2.463
Osteoporotic (<-2.5 SD)** *Reference Level: normal BMD	1.69	0.233; 12.253

### Vertebral fractures

**Figure 2:**
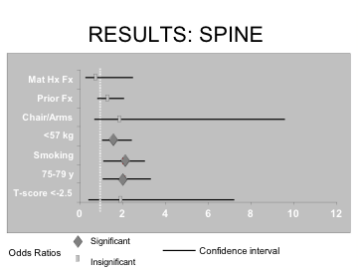
Odds ratios and confidence intervals for each risk factor that predicts higher risk of vertebral fracture.

The results of multivariate logistic regression analyses for each of the seven risk factors thought to predict a new vertebral fracture within the CANDOO database are depicted in Figure 2 and Table 3. Of all the risk factors, three were found to be significant predictors of new spinal fractures. Current smokers and those between the ages of 75–79 years were approximately two times more likely to develop a new vertebral fracture. Individuals weighing less than 57 kg were about 1.6 times more likely to develop a new fracture at this site. The other four risk factors did not reach statistical significance in terms of their predictability of a new fracture at vertebral sites in CANDOO.

**Table 3:** Vertebral Fracture Results Odds ratios and 95% confidence intervals point estimates for each risk factor in the development of a new vertebral fracture

**RISK FACTOR**	**Odds Ratio**	**95% Confidence Interval**
Weight (< 57 Kg) *Reference Level: ≥ 57 Kg	1.57	1.035; 2.373
Use of Arms to Rise *Reference Level: rise without arms	1.72	0.981; 3.023
Smoking Status *Reference Level: no smoking	1.95	1.199; 3.184
Age (65 to 69 y) *Reference Level: < 65	1.36	0.805; 2.309
Age (70–74 y) *Reference Level: < 65	1.28	0.753; 2.174
Age (75–79 y) *Reference Level: < 65	1.96	1.096; 3.508
Age (80–84 y) *Reference Level: < 65	0.93	0.368; 2.334
Age (85+y) *Reference Level: < 65	1.32	0.375; 4.631
Previous Fracture After 50 y *Reference Level: no fracture after 50	1.37	0.931; 2.012
Maternal History of Fracture *Reference Level: no family history	0.85	0.296; 2.412
Osteoporotic (<-2.5 SD)** *Reference Level: normal BMD	1.85	0.448; 7.676

### Wrist fractures

**Figure 3:**
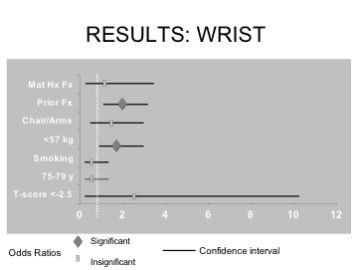
Odds ratios and confidence intervals for each risk factor that predicts higher risk of wrist fracture.

Table 4 shows odds ratios and 95% confidence intervals for each risk factor for fractures at the wrist. Each of the seven risk factors' independent ability to predict a new wrist fracture is illustrated in Figure 3. Prior fracture after the age of 50 and a body weight less than 57 kg were deemed significant risk factors for a new incident wrist fracture. Individuals with a prior fracture after the age of 50 years and those weighing less than 57 kg were about two and 1.7 times more likely to develop a new wrist fracture, respectively. The other five risk factors were not statistically significant.

**Table 4:** Wrist Fracture Results Odds ratios and 95% confidence intervals point estimates for each risk factor in the development of a new fracture at the wrist

**RISK FACTOR**	**Odds Ratio**	**95% Confidence Interval**
Weight (< 57 Kg) *Reference Level: ≥ 57 Kg	1.71	1.007; 2.897
Use of Arms to Rise *Reference Level: rise without arms	1.53	0.790; 2.981
Smoking Status *Reference Level: no smoking	0.62	0.259; 1.466
Age (65 to 69 y) *Reference Level: < 65	0.76	0.387; 1.485
Age (70–74 y) *Reference Level: < 65	1.00	0.539; 1.843
Age (75–79 y) *Reference Level: < 65	0.57	0.238; 1.387
Age (80–84 y) *Reference Level: < 65	0.46	0.134; 1.603
Age (85+y) *Reference Level: < 65	0.83	0.183; 3.776
Previous Fracture After 50 y *Reference Level: no fracture after 50	1.96	1.191; 3.223
Maternal History of Fracture *Reference Level: no family history	1.25	0.430; 3.627
Osteoporotic (<-2.5 SD)** *Reference Level: normal BMD	2.26	0.476; 10.682

### Rib fractures

**Figure 4:**
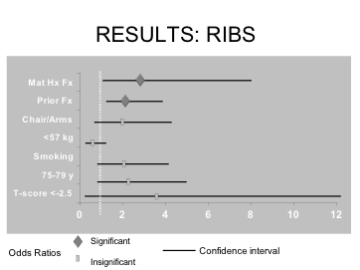
Odds ratios and confidence intervals for each risk factor that predicts higher risk rib fracture.

Individuals with a maternal history of a hip fracture and a prior fracture after the age of 50 years were approximately three and two times more likely, respectively, to develop a new rib fracture during the course of the study. Those with any of the other five risk factors were not at significantly increased risk of sustaining a new fracture at the rib. These data are displayed in Table 5 and Figure 4.

**Table 5:** Rib Fracture Results Odds ratios and 95% confidence intervals point estimates for each risk factor in the development of a new fracture at the rib

**RISK FACTOR**	**Odds Ratio**	**95% Confidence Interval**
Weight (< 57 Kg) *Reference Level: ≥ 57 Kg	0.47	0.222; 1.011
Use of Arms to Rise *Reference Level: rise without arms	1.93	0.868; 4.272
Smoking Status *Reference Level: no smoking	1.94	0.903; 4.179
Age (65 to 69 y) *Reference Level: < 65	1.42	0.631; 3.210
Age (70–74 y) *Reference Level: < 65	1.58	0.709; 3.516
Age (75–79 y) *Reference Level: < 65	2.26	0.928; 5.486
Age (80–84 y) *Reference Level: < 65	2.69	0.950; 7.625
Age (85+y) *Reference Level: < 65	1.04	0.126; 8.478
Previous Fracture After 50 y *Reference Level: no fracture after 50	2.16	1.201; 3.874
Maternal History of Fracture *Reference Level: no family history	2.89	1.035; 8.081
Osteoporotic (<-2.5 SD)** *Reference Level: normal BMD	3.89	0.367; 41.263

## Discussion

Even with the advent of the recent publication of the 2002 Osteoporosis Society of Canada's Clinical Practice Guidelines, there remains no universal way of identifying those with osteoporosis and those at increased risk of fracture. The clinical goal must be to identify the substantial percentage of the population at high risk for fracture while simultaneously limiting any unnecessary testing to the increasingly burdened health care system [16,17]. Prevention of an incident fracture and the cascade of subsequent fractures is the ultimate objective.

We found a variety of clinical risk factors in conjunction with BMD to be extremely helpful in distinguishing those at high risk of fracture in CANDOO.

The association of increasing age with declining bone density has long been recognized as being the predominant risk factor for fracture. Age was shown to be an independent predictor of hip fracture in the EPIDOS study [6]. For ages 45 through 85, the ten year probability of a fracture in the forearm, humerus, spine or hip increases five times in men and eight times in women [18]. It has been estimated by Kanis et al. that risk of fracture in the forearm alone can increase eight times between the ages of 45 and 85 [18]. The results from the CANDOO database showed that although age, especially between 75 to 79 years, was associated with heightened risk of fracture at all sites, it only reached significance for predicting future fractures at vertebral sites. These results indicate that it is possible that age alone may not be definitive in predicting overall fracture risk.

In a clinical setting, BMD remains the gold-standard in assessing fracture risk so long as it is considered within the context of age. Cummings et al. showed that for each standard deviation decline in femoral neck BMD is associated with 2.6 times the risk of hip fracture in postmenopausal women aged 65 years or more [19]. Although BMD can identify people who are at increased risk of experiencing a fracture, it cannot, with any certainty, identify those individuals who will necessarily sustain a future fracture. Moreover, BMD testing is both inconvenient and expensive. Many under-serviced areas do not have the technology available for assessing BMD and even in those areas where machines are available, such procedures may be difficult to access due to mobility issues for osteoporotic patients and elderly subjects in long-term care facilities. Results from the CANDOO database did not indicate the overwhelming importance of BMD values in predicting those at increased fracture risk at any of the four sites considered. While a clinically relevant association was seen between BMD and fracture, its association was not statistically significant. This could at least in part arise from the small number of eligible subjects in CANDOO with BMD assessments.

Previous fracture history is well known to be predictive of future fracture risk [10,20-23]. The number of prior fractures at the site of incident fracture (i.e. hip, spine) combined with age has been shown to increase fracture risk 1.5 to 9.5 times [10,20,21,23-27]. Prior fracture at vertebral sites increases the risk of future fracture by as much as four times [28,29]. A previous history of fracture represented an important factor in evaluating the risk of future non-vertebral (wrist and rib) fractures in the CANDOO patients. At hip related sites within the CANDOO population, previous fracture history was not significant in predicting future hip fracture risk. This may be explained by the low incidence of hip fractures and the fact that many vertebral fractures remain undiagnosed. In fact, it has been estimated that less than one third of all vertebral fractures are clinically diagnosed [30].

Much evidence has indicated that those with a maternal history of fracture, especially at the hip, are at increased risk of future fracture. Moreover, those whose maternal history involves a grandmother, carry an even greater risk of hip fracture [11]. Within the CANDOO patients, the predictive ability of this variable appeared to be less important. At all sites, with the exception of the rib, maternal family history of fracture showed no significant association with increased fracture risk. This could possibly be explained by poor recollection or documentation of maternal history within the baseline assessment.

Low body weight was shown to be a significant predictor of future fracture at both vertebral and wrist sites. It played a significant role in assessing fracture risk at these sites, but appeared to be less important in determining risk of future fracture in the hip or rib.

The direct effects of smoking resulting in declining BMD and increased fracture risk have been identified by a variety of different studies [31-34]. Individuals who reported smoking in CANDOO were only at significantly increased risk of fracture at vertebral sites. These results indicate that at least in CANDOO, smoking as a risk factor is limited at other sites.

Many risk factors incorporated into the Fracture Index and other publications focus on biological, historical or BMD-affecting variables in risk factor analysis. Although the importance of these particular risk factors is unquestionable, a considerable number of osteoporotic fractures result from falls. Easily assessed neuromuscular measures of fall-related hip fracture have been examined in a few studies [10,35,36]. With the use of data from EPIDOS, Dargent-Molina et al. found that four predictors of fall-related fracture (slow gait speed, difficulty in tandem walking, reduced visual acuity, small calf circumference) were significantly associated with increased risk of future hip fracture [35]. A similar study confirmed that a simple and efficient measure of gait speed had the same discriminant value for fracture prediction as femoral BMD at all cutoff values [36]. It was the prospective study by Cummings et al. [10] that indicated the importance of examining one's ability to rise from a chair without the use of arms in risk assessment. They determined that this factor alone was the most significant at predicting hip fracture and that the addition of other neuromuscular assessment tests added little to the prediction of subsequent hip fracture [10].

The use of arms to rise from a seated position was found to be the single most important predictor of increased fracture risk at the hip in CANDOO patients. These results indicate that both research and clinical settings should place a greater emphasis than is currently standard procedure on this variable or others that examine neuromuscular ability. Such an easily assessed risk factor would make for an efficient and effective mechanism to gauge risk across the entire population, especially in those for which BMD testing is not feasible.

Many of these risk factors have been successfully incorporated into clinical risk assessment tools, while others such as the use of arms to rise from a chair tend to be ignored. A variety of current risk assessment mechanisms are used routinely in clinical practice, including the Osteoporosis Risk Assessment Instrument (ORAI), Simple Calculated Osteoporosis Risk Estimation (SCORE) and the module Physicians' Information and Education Resource (PIER) [37-39].

The ORAI is a clinical assessment tool that was designed to identify women over the age of 45 who are at increased risk for osteoporosis who should undergo BMD testing. This simple tool uses three items (age, weight and estrogen use) to gauge risk and has been validated to identify over 90% of women at increased risk of osteoporotic fracture while ensuring that less than 50 % of those with normal BMD are selected [37]. Although this procedure has proven to be useful in clinically identifying patients at risk, it only allows for examination of females and relies completely on historical risk factors.

A similar mechanism for identifying individuals at increased risk in clinical practice was set forth in SCORE. This tool relies on six risk factors rather than three in order to assess risk of osteoporotic fracture. These six variables include: race, age, rheumatoid arthritis, history of non-traumatic fracture after age 45, weight and estrogen use [37]. Despite its incorporation of more risk factors, SCORE has been shown to have similar sensitivity to ORAI but greater selection of individuals with normal BMD [40].

The effectiveness of determining those individuals at increased risk of future fracture not only revolves around public awareness of risk factors, but relies heavily on the ability of physicians (especially in primary care settings) to effectively and efficiently identify at-risk individuals. Module PIER [39] is a web and computer based resource designed to guide physicians through the diagnosis, treatment and management of a plethora of diseases including osteoporosis. Their recommendations include the use of the Fracture Index for risk assessment and analyses of other variables including use of corticosteroid therapy for more than 3 months, impaired vision, low calcium intake, low physical activity, dementia, alcohol consumption of greater than 2 drinks per day and estrogen deficiency before 45 years of age. Subsequently, any post-menopausal woman over 65 years of age or those under 65 who have at least one of these risk factors is recommended to have her BMD assessed. The inclusion of risk factors to assess frailty and mobility in PIER, such as the use of arms to rise from a chair and impaired vision, takes into consideration the importance of preventing falls and future fracture rather than solely basing risk assessment on historical risk factors.

Our study incorporated a large sample of post-menopausal women from the CANDOO database. The subject data from this database were homogeneous as they stemmed from a group of patients who were assessed in the tertiary care setting and represented a "real world" group [13]. Moreover, the multivariable analyses involved detailed and controlled delineation of potential confounding variables.

Although the study design attempted to minimize limiting factors, it should be recognized that much of the information in the CANDOO database relied on factual recall from patients, some of which was reviewed at considerable length after the experience had occurred. In addition, although careful delineation of confounding variables was carried out, it remains uniquely possible that other potential confounders played a role. Assessment of fractures for the CANDOO database were either confirmed or self-reported and as a result sub-clinical fractures in patients could have been missed. Also, the lack of data on male patients meant only females were assessed and, as such, results should not necessarily be extrapolated to the male population.

Further studies will need to be undertaken in attempt to identify a more definitive and indicative group of risk factors that can be used across genders and populations to assess future fracture risk and the subsequent development of osteoporosis.

## Conclusion

Despite extensive research and new treatment strategies, osteoporosis remains one of the "sleeping giants of health care". Much of the population remains undiagnosed and unaware of the importance of early recognition and preventative treatment. As the "boomer bulge" continues to progress through middle age and into senior brackets, the effects of osteoporosis on both the individual and health care system will be enormous. This study has shown that there exists a variety of predictors of future fracture, besides BMD, that can be easily assessed by a physician. The predictive significance of each of these risk factors has been shown to be dependent on the site of incident fracture. The assessment of unconventional risk factors such as examining one's ability to rise from a chair without the use of arms to gauge proprioception, strength and coordination are simple, convenient and valuable in terms of predicting fracture risk. These risk assessment factors can be easily incorporated into routine clinical practice, especially where BMD testing is unfeasible. A better understanding of which factors lead to an increase in the incidence of fractures is a crucial step in evaluating patients at risk and designing therapeutic strategies.

## Competing interests

The author(s) declare that they have no competing interests.

## Authors' contributions

RB- conceived design, participated in analysis and coordination and drafted manuscript

JDA- conceived design, participated in CANDOO database, participated in analysis and manuscript

AP- conceived design, participated in CANDOO database, coordinated analysis and participated in manuscript

GI- performed statistical analysis and participated in study design

WO- participated in CANDOO, study design, and analysis

RS- participated in CANDOO, study design, and analysis

TM- participated in CANDOO, study design, and analysis

RJ- participated in CANDOO, study design, and analysis

JB- participated in CANDOO, study design, and analysis

DH- participated in CANDOO, study design, and analysis

AP- participated in CANDOO, study design, and analysis

MP- participated in CANDOO, study design, and analysis

CG- participated in statistical analysis and study design

## Pre-publication history

The pre-publication history for this paper can be accessed here:


